# Impact of the COVID-19 Pandemic on Trauma Encounters

**DOI:** 10.1177/00031348211029858

**Published:** 2023-03

**Authors:** Nicholas W. Sheets, Oluwatosin S. Fawibe, Ahmed Mahmoud, Bhani Chawla-Kondal, Napatkamon Ayutyanont, David S. Plurad

**Affiliations:** 1 43951 Riverside Community Hospital, Riverside, CA, USA

**Keywords:** special topics, trauma, trauma acute care

## Abstract

**Objectives:**

The Coronavirus Disease 2019 pandemic has affected the health care system significantly. We compare 2019 to 2020 to evaluate how trauma encounters has changed during the pandemic.

**Methods:**

Retrospective analysis using a large US health care system to compare trauma demographics, volumes, mechanisms of injury, and outcomes. Statistical analysis was used to evaluate for significant differences comparing 2019 to 2020.

**Results:**

Data was collected from 88 hospitals across 18 states. 169 892 patients were included in the study. There were 6.3% fewer trauma patient encounters in 2020 compared to 2019. Mechanism of injury was significantly different between 2019 and 2020 with less blunt injuries (89.64% vs. 88.39%, *P* < .001), more burn injuries (1.84% vs. 2.00%, *P* = .021), and more penetrating injuries (8.58% vs. 9.75%, *P* < .001). Compared to 2019, patients in 2020 had higher mortality (2.62% vs. 2.88%, *P* < .001), and longer hospital LOS (3.92 ± 6.90 vs. 4.06 ± 6.56, *P* < .001).

**Conclusion:**

The COVID-19 pandemic has significantly affected trauma patient demographics, LOS, mechanism of injury, and mortality.

## Introduction

The Coronavirus Disease 2019 (COVID-19) pandemic has significantly affected the United States health care system including trauma centers and patients.^1-3^ During the initial months of the pandemic, domestic violence calls have increased by 25% while traffic congestion has diminished accompanying a >60% decrease in local travel in the United States.^4-6^ While some areas were seeing decreases in motor vehicle accidents, motor vehicle fatalities were increasing likely from the public driving faster on highways.^7,8^ A pandemic of this magnitude is rare and provides a critical and unique time to evaluate trends in trauma. Previous studies have shown decreases in trauma admissions and increases in penetrating and violence related trauma.^9-16^ Evaluation of the United States traumatic injuries during 2020 is warranted to evaluate how fluctuations has changed during a unique period in history and help anticipate future trends during similar situations.

## Methods

Monthly trauma volumes from 88 hospitals in 18 states were queried from a large US health care system database from January 2019 to December 2020 identifying 172,061 patients. Patients with missing data on gender (n = 371), Injury Severity Score (ISS) (n = 202), not having admission dates from January 2019 to December 2020 (n = 21), and missing ICD-10 codes on mechanism of injury (n = 1575) were excluded. A total of 169 892 patients with 173 936 trauma encounters were included to compare monthly trauma volumes in the pre-pandemic period (January to December 2019) to the pandemic period (January to December 2020). Data on age, sex, race/ethnicity, hospital length of stay (LOS), ISS, and discharge description were extracted. Mechanism of injury was classified based on the ICD-10 codes. Categorical variables were reported as percentages and continuous variables reported as means. Chi-square tests were used to compare categorical variables and two-sample t-test was used to compare continuous variables. *P*-value threshold of *P* < .05 was used for 2-tailed tests. The study was approved by the hospitals Institutional Review Board. Data analysis was performed using SAS 9.4 and R version 4.0.2.

## Results

Data was collected from 88 hospitals across 18 states (CA, CO, FL, GA, ID, IN, KS, KY, LA, MO, MS, NH, NV, SC, TN, TX, UT, VA). 169 892 trauma patients were included in the study. There were 89 813 patients in 2019 and 84 123 patients in 2020 (Table 1). Trauma patients in 2020 were more likely to be male (56.60% vs. 57.44%, *P* < .001), African American or other race (10.45% vs. 10.96%, *P* < .001; 5.15% vs. 5.70% *P* < .001), more severely injured by ISS >12 (17.25% vs. 19.53% *P* < .001), have a higher mortality (2.62% vs. 2.88%, *P* < .001), and longer hospital LOS (3.92 ± 6.90 vs. 4.06 ± 6.56, *P* < .001).

**Table 1.** Trauma Characteristics Comparing 2019 to 2020.

Trauma patient characteristics	2019	2020	*P* value
	Mean ± SD/n (%)	Mean ± SD/n (%)	
No of encounters	89 813	84 123	
No of patients	87 007	82 885	
Age (mean)	53.87 ± 24.01	53.87 ± 23.84	.985
Male (%)	49 243 (56.60%)	47 606 (57.44%)	**<.001**
Race			
White	64 394 (74.01%)	60 665 (73.19%)	**.010**
African American	9094 (10.45%)	9084 (10.96%)	**<.001**
Asian	1601 (1.84%)	1469 (1.77%)	.356
Hispanic	7436 (8.55%)	6942 (8.38%)	.309
Other	4482 (5.15%)	4725 (5.70%)	**<.001**
ISS (mean)	8.17 ± 7.50	8.72 ± 7.63	**<.001**
ISS ≤ 12	71 998 (82.75%)	66 697 (80.47%)	**<.001**
ISS > 12	15 009 (17.25%)	16 188 (19.53%)	**<.001**
Mechanism of injury			
Blunt	77 992 (89.64%)	73 258 (88.39%)	**<.001**
Burn	1603 (1.84%)	1655 (2.00%)	**.021**
Penetrating	7469 (8.58%)	8081 (9.75%)	**<.001**
LOS (mean)	3.92 ± 6.90	4.06 ± 6.56	**<.001**
Discharge information			
Mortality	2279 (2.62%)	2383 (2.88%)	**.001**
Home	52 974 (60.88%)	50 462 (60.88%)	.994
Hospice	1290 (1.48%)	1495 (1.80%)	**<.001**
Facility/other hospitals	5307 (6.10%)	5640 (6.80%)	**<.001**
Rehab	21 209 (24.38%)	19 366 (23.36%)	**<.001**
Others	3948 (4.54%)	3539 (4.27%)	**.007**

Significant *P*-values bolded (*P* < .05).

Abbreviations: ISS, injury severity score; LOS, length of stay.

**Figure 1.**
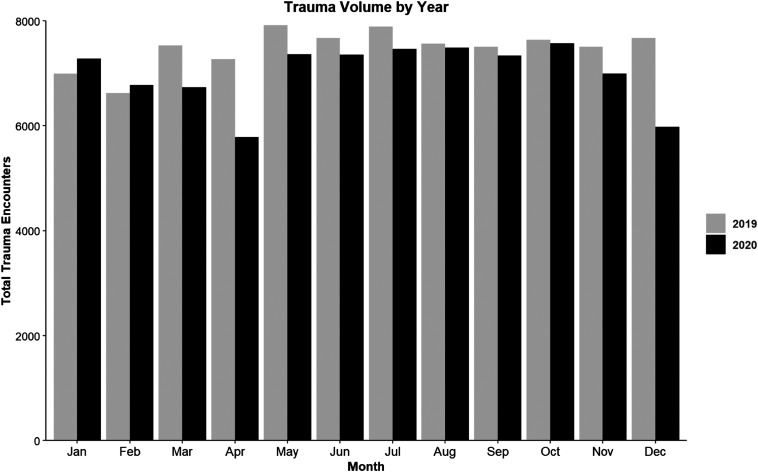
Trauma volume by month comparing 2019 to 2020.

Compared to 2019, there was a 6.3% decrease in volume in 2020 with a decrease in volume beginning in March of 2020 that persisted the remainder of the year with a gradual increase to pre-COVID volumes (Figure 1). The initial 20.5% decrease in trauma volumes occurred in April 2020. A second decrease in trauma volume occurs in November 2020 with a nadir 22.1% decrease in trauma volume in December 2020.

**Figure 2.**
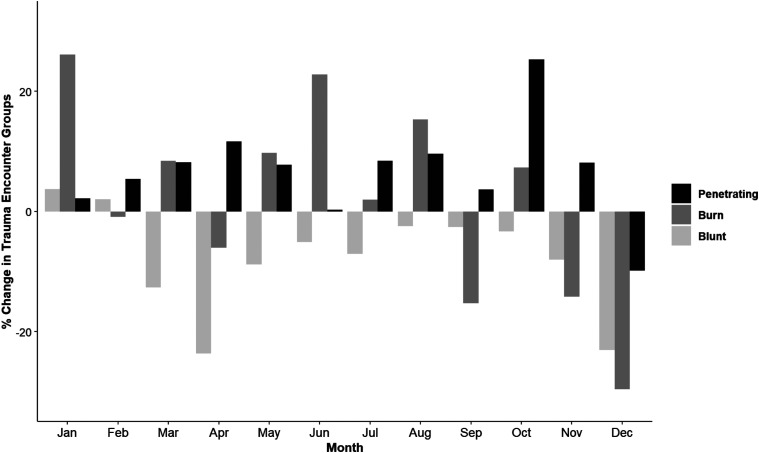
Trauma mechanism comparing 2019 to 2020.

Significant changes in mechanism of injury and ISS begin in March (Table 2). Mechanism of injury are significantly different between 2019 and 2020 with less blunt injuries (89.64% vs. 88.39%, *P* < .001), more burn injuries (1.84% vs. 2.00%, *P* = .021), and more penetrating injuries (8.58% vs. 9.75%, *P* < .001). Penetrating trauma remains increased for 2020 compared to 2019 until December where there is a percentage decrease in all mechanisms of injury (Figure 2).

**Table 2.** Trauma Characteristics by Month in 2019 and 2020.

	January			Feb			March			April			May			June		
	2019	2020	*P*	2019	2020	*P*	2019	2020	*P*	2019	2020	*P*	2019	2020	*P*	2019	2020	*P*
	Mean ± SD/n (%)	Mean ± SD/n (%)		Mean ± SD/n (%)	Mean ± SD/n (%)		Mean ± SD/n (%)	Mean ± SD/n (%)		Mean ± SD/n (%)	Mean ± SD/n (%)		Mean ± SD/n (%)	Mean ± SD/n (%)		Mean ± SD/n (%)	Mean ± SD/n (%)	
Total encounters	6995	7276		6624	6772		7534	6735		7272	5784		7920	7364		7674	7356	
No of patients	6704	7116		6358	6633		7285	6585		7006	5657		7672	7229		7454	7239	
Age (mean)	56.54 ± 23.81	55.89 ± 23.98	.105	56.16 ± 23.63	56.44 ± 23.68	.488	54.52 ± 23.87	54.80 ± 23.83	.481	54.06 ± 24.42	53.80 ± 24.04	.552	53.31 ± 24.16	53.36 ± 23.79	.896	53.36 ± 23.82	52.68 ± 23.81	.079
Male (%)	3858 (55.15%)	934 (54.07%)	.199	3598 (54.32%)	3648 (53.87%)	.614	4272 (56.70%)	3840 (57.02%)	.719	4083 (56.15%)	3402 (58.82%)	**.002**	4527 (57.16%)	4306 (58.47%)	.104	4351 (56.70%)	4309 (58.58%)	**.021**
ISS (mean)	8.30 ± 7.67	8.38 ± 7.41	.557	8.35 ± 7.70	8.42 ± 7.11	.550	8.33 ± 7.69	8.81 ± 7.60	**<.001**	8.27 ± 7.57	9.00 ± 7.82	**<.001**	8.28 ± 7.76	8.74 ± 7.80	**<.001**	8.24 ± 7.56	8.85 ± 8.02	**<.001**
ISS > 12	1271 (18.17%)	1287 (17.69%)	.466	1180 (17.81%)	1220 (18.02%)	.778	1382 (18.34%)	1344 (19.96%)	**.015**	1253 (17.23%)	1203 (20.80%)	**<.001**	1402 (17.70%)	1443 (19.60%)	**.003**	1374 (17.90%)	1488 (20.23%)	**<.001**
LOS (mean)	4.08 ± 6.15	4.17 ± 7.52	.417	4.11 ± 7.59	4.23 ± 6.77	.342	4.03 ± 7.26	3.93 ± 6.57	.403	3.81 ± 7.62	3.89 ± 6.66	.531	3.80 ± 6.38	3.90 ± 6.53	.353	3.78 ± 6.59	4.05 ± 6.44	**.011**
Mortality	181 (2.59%)	178 (2.45%)	.628	188 (2.84%)	146 (2.16%)	**.013**	199 (2.64%)	206 (3.06%)	.148	186 (2.56%)	180 (3.11%)	.064	203 (2.56%)	181 (2.46%)	.716	169 (2.20%)	214 (2.91%)	**.007**
Mechanism of injury																		
Blunt	6276 (89.72%)	6509 (89.46%)	.627	6016 (90.82%)	6138 (90.64%)	.737	6805 (90.32%)	5946 (88.29%)	**<.001**	6557 (90.17%)	5006 (86.55%)	**<.001**	7068 (89.24%)	6443 (87.49%)	**.001**	6843 (89.17%)	6494 (88.28%)	.089
Burn	134 (1.92%)	169 (2.32%)	.103	111 (1.68%)	110 (1.62%)	.868	131 (1.74%)	142 (2.11%)	.122	116 (1.60%)	109 (1.88%)	.232	144 (1.82%)	158 (2.15%)	.163	127 (1.65%)	156 (2.12%)	**.041**
Penetrating	585 (8.36%)	598 (8.22%)	.778	497 (7.50%)	524 (7.74%)	.632	598 (7.94%)	647 (9.61%)	**<.001**	599 (8.24%)	669 (11.57%)	**<.001**	708 (8.94%)	763 (10.36%)	**.003**	704 (9.17%)	706 (9.60%)	.388
Race																		
White	5368 (76.74%)	5450 (74.90%)	**.028**	5030 (75.94%)	5064 (74.78%)	.200	5588 (74.17%)	4960 (73.65%)	.932	5412 (74.42%)	4226 (73.06%)	.233	5845 (73.80%)	5422 (73.63%)	.798	5708 (74.38%)	5323 (72.36%)	**.008**
African American	687 (9.82%)	722 (9.92%)	.801	613 (9.25%)	630 (9.30%)	.904	799 (10.85%)	717 (10.96%)	.859	738 (10.36%)	641 (11.38%)	.069	829 (10.72%)	768 (10.73%)	1.000	816 (10.88%)	863 (12.03%)	**.031**
Asian	121 (1.73%)	150 (2.06%)	.155	105 (1.59%)	131 (1.93%)	.135	162 (2.20%)	115 (1.76%)	.071	106 (1.46%)	78 (1.35%)	.682	148 (1.91%)	113 (1.58%)	.136	120 (1.60%)	120 (1.67%)	.778
Hispanic	522 (7.46%)	620 (8.52%)	**.018**	543 (8.20%)	585 (8.64%)	.347	598 (8.12%)	564 (8.62%)	.302	648 (9.10%)	490 (8.70%)	.455	685 (8.86%)	610 (8.52%)	.490	635 (8.47%)	627 (8.74%)	.575
Others	297 (4.25%)	334 (4.59%)	.337	333 (5.03%)	362 (5.35%)	.429	387 (5.14%)	379 (5.63%)	.207	368 (5.06%)	349 (6.03%)	**.017**	413 (5.21%)	451 (6.12%)	**.016**	395 (5.15%)	423 (5.75%)	.111

	July			August			September			October			November			December		
	2019	2020	*P*	2019	2020	*P*	2019	2020	*P*	2019	2020	*P*	2019	2020	*P*	2019	2020	*P*
	Mean ± SD/n (%)	Mean ± SD/n (%)		Mean ± SD/n (%)	Mean ± SD/n (%)		Mean ± SD/n (%)	Mean ± SD/n (%)		Mean ± SD/n (%)	Mean ± SD/n (%)		Mean ± SD/n (%)	Mean ± SD/n (%)		Mean ± SD/n (%)	Mean ± SD/n (%)	
Total encounters	7896	7468		7569	7487		7504	7335		7644	7574		7505	6995		7676	5977	
No of patients	7656	7361		7340	7396		7300	7254		7447	7508		7293	6946		7492	5961	
Age (mean)	52.94 ± 24.01	52.39 ± 23.68	.153	53.43 ± 24.03	53.03 ± 23.60	.306	53.86 ± 23.73	53.41 ± 23.86	.253	54.03 ± 24.34	53.47 ± 24.03	.158	54.86 ± 23.94	55.21 ± 23.67	.371	55.88 ± 23.98	55.44 ± 23.80	.293
Male (%)	4465 (56.55%)	4401 (58.93%)	**.003**	4340 (57.34%)	4380 (58.50%)	.153	4246 (56.58%)	4248 (57.91%)	.105	4361 (57.05%)	4375 (57.76%)	.383	4261 (56.78%)	3951 (56.48%)	.735	4143 (53.97%)	3384 (56.62%)	**.002**
ISS (mean)	8.08 ± 7.24	8.96 ± 7.84	**<.001**	8.19 ± 7.57	8.88 ± 7.91	**<.001**	8.31 ± 7.49	8.76 ± 7.62	**<.001**	8.39 ± 7.43	8.71 ± 7.36	**.007**	8.29 ± 7.15	8.99 ± 7.69	**<.001**	8.41 ± 7.42	8.86 ± 7.51	**.001**
ISS > 12	1379 (17.46%)	1565 (20.96%)	**<.001**	1319 (17.43%)	1540 (20.57%)	**<.001**	1362 (18.15%)	1462 (19.93%)	**.006**	1392 (18.21%)	1503 (19.84%)	**.011**	1341 (17.87%)	1446 (20.67%)	**<.001**	1372 (17.87%)	1190 (19.91%)	**.003**
LOS (mean)	3.78 ± 7.17	4.01 ± 6.90	**.043**	3.83 ± 7.90	4.12 ± 6.69	**.015**	3.89 ± 6.36	4.15 ± 6.57	**.015**	3.96 ± 6.17	4.13 ± 6.28	.088	4.08 ± 6.55	4.12 ± 5.76	.691	4.00 ± 6.60	3.96 ± 5.24	.673
Mortality	178 (2.25%)	206 (2.76%)	.051	176 (2.33%)	223 (2.98%)	**.015**	174 (2.32%)	211 (2.88%)	**.037**	215 (2.81%)	208 (2.75%)	.842	187 (2.49%)	217 (3.10%)	**.029**	223 (2.91%)	213 (3.56%)	**.034**
Mechanism of injury																		
Blunt	6966 (88.22%)	6473 (86.68%)	**.004**	6776 (89.52%)	6610 (88.29%)	**.017**	6685 (89.09%)	6512 (88.78%)	.570	6916 (90.48%)	6684 (88.25%)	**<.001**	6770 (90.21%)	6227 (89.02%)	**.021**	6941 (90.42%)	5339 (89.33%)	**.037**
Burn	207 (2.62%)	211 (2.83%)	.468	137 (1.81%)	158 (2.11%)	.204	137 (1.83%)	116 (1.58%)	.278	123 (1.61%)	132 (1.74%)	.562	120 (1.60%)	103 (1.47%)	.582	125 (1.63%)	88 (1.47%)	.509
Penetrating	723 (9.16%)	784 (10.50%)	**.006**	656 (8.67%)	719 (9.60%)	**.049**	682 (9.09%)	707 (9.64%)	.262	605 (7.91%)	758 (10.01%)	**<.001**	615 (8.19%)	665 (9.51%)	**.006**	610 (7.95%)	550 (9.20%)	**.010**
Race																		
White	5849 (74.08%)	5386 (72.12%)	.095	5557 (75.15%)	5476 (75.01%)	.869	5572 (74.25%)	5334 (72.72%)	.080	5684 (74.36%)	5501 (72.63%)	**.028**	5578 (76.06%)	5211 (76.28%)	.765	5651 (75.25%)	4399 (75.58%)	.668
African American	854 (10.82%)	883 (11.82%)	**.028**	803 (10.86%)	817 (11.19%)	.536	773 (10.49%)	882 (12.30%)	**.001**	784 (10.45%)	900 (12.14%)	**.001**	758 (10.34%)	720 (10.54%)	.711	804 (10.71%)	597 (10.26%)	.419
Asian	130 (1.65%)	136 (1.82%)	.391	153 (2.07%)	127 (1.74%)	.162	142 (1.93%)	111 (1.55%)	.091	138 (1.84%)	161 (2.17%)	.165	149 (2.03%)	125 (1.83%)	.418	154 (2.05%)	120 (2.06%)	1.000
Hispanic	675 (8.73%)	591 (8.17%)	.230	653 (8.83%)	639 (8.75%)	.892	665 (9.03%)	605 (8.44%)	.217	671 (8.95%)	618 (8.34%)	.196	619 (8.44%)	538 (7.88%)	.232	647 (8.62%)	512 (8.80%)	.735
Others	388 (4.91%)	472 (6.32%)	**<.001**	403 (5.32%)	428 (5.72%)	.309	352 (4.69%)	403 (5.49%)	**.029**	367 (4.80%)	394 (5.20%)	.273	401 (5.34%)	401 (5.73%)	.323	420 (5.47%)	349 (5.84%)	.375

Significant *P*-values bolded (*P* < .05).

Abbreviations: ISS, injury severity score; LOS, length of stay.

Patients in 2020 had a longer hospital LOS in days (3.92 ± 6.90 vs. 4.06 ± 6.56, *P* < .001). This significant difference appeared from June to September. There was a higher percentage mortality (2.62% vs. 2.88%, *P* = .001) and patients discharged to hospice (1.48% vs. 1.80%, *P* < .001) in 2020 compared to 2019.

## Discussion

Since the COVID-19 pandemic, trauma volumes of hospitals have substantially decreased. This is suspected to be largely due to the regulations and behavioral changes among the public. Our study showed that within the 2020 COVID-19 pandemic, an approximately 6% cumulative decrease in trauma volume has occurred with the largest decreases occurring in April and December 2020. This decrease begins in March, possibly corresponding with stay-at-home orders. This mirrors other studies showing decreased trauma volumes across the United States and among other countries. Kamine et al^17^ showed that trauma volumes decreased 57.4% during February to April 2020 in comparison to previous years in their Level II trauma hospital in New Hampshire. Sherman et al showed decreased trauma volumes by 70% at a Level I trauma center in Louisiana during the pandemic during March to May 2020.^18^ Qasim et al found a 20.3% decrease in trauma volume in Philadelphia during March to May 2020.^19^ Matthay et al^16^ found a 50% decrease in trauma volume after the stay-at-home order from March to June 2020 in comparison to the previous months in San Francisco. Similar drops in trauma volume occurred in Los Angeles CA during January to June 2020 while a return to pre pandemic volumes occur.^20^ While most studies evaluate the early pandemic up to the first 6 months, our study evaluates the entirety of 2020 as the decrease in trauma volume becomes less significant as the year progresses. A second inflection in trauma volume occurs in November 2020, likely corresponding to the third and largest wave of COVID-19 positivity.^21^ Past epidemics have seen similar changes. In the previous 2003 SARS epidemic in Taiwan, emergency departments visits decreased by 51% and trauma visits decreased by 57.6% but recovered to pre-epidemic numbers in July, the same month that the World Health Organization removed Taiwan from the list of SARS epidemic countries.^22,23^

This study found an increase percentage of penetrating trauma that persisted for the pandemic. Many other studies regarding trauma have found similar increases in penetrating trauma. Southern California^24^ penetrating trauma increased from 10.3% to 13.0%, A Los Angeles County study^20^ separately identified increase in penetrating trauma 15.4% to 15.7%, Philadelphia^19^ penetrating trauma increased 17.5% to 23.7%, and San Francisco^16^ violence related injuries increased 17% to 46%. This study identified a concurrent decrease in blunt trauma accompanying the increased proportions of penetrating trauma which is similar to previous studies. This may suggest that while stay at home orders may affect blunt trauma such as motor vehicle accidents, violence related injuries continue and may be exacerbated by socioeconomic stressors inflicted by the pandemic. A second inflection in trauma volumes and mechanism of injury occurs in November 2020 during the height of the third wave of positive COVID-19 cases. This decrease in volume by 22.1% in December accompanies a decrease in all mechanisms of injury; 23.1% blunt, 29.6% in burns, and 9.8% penetrating. This is the first month during the pandemic where penetrating trauma has decreased. Changes in trauma volume and mechanism during the beginning of 2020 may be attributed to stay-at-home orders while the second decrease may be from other causes and require further investigation.

There were significant changes in hospital LOS and mortality in 2020. The difference in hospital LOS is relatively small and coincides with no difference in ICU LOS found in previous reports.^20^ While mortality differences fluctuated throughout the year, the greatest mortality among trauma patients in 2020 occurred in March (3.06%) and April (3.11%), then again in November (3.10%) and December (3.56%). The peaks in mortality occur during the initial stay-at-home orders and the third peak of COVID-19 positivity yet the explanation for this change in mortality is difficult to ascertain. Kaufman et al^25^ found that 2.6% of trauma patients tested positive for COVID-19 and had an increased risk of death (OR 6.05, 95% CI 2.29, 15.99) when matched with COVID-19 negative trauma patients. Conversely, Ghafil et al^20^ found no difference in mortality during the COVID-19 pandemic. Further studies regarding the change in outcomes during the COVID-19 pandemic are warranted as patient and health care system factors may help explain these differences.

Limitations present in this study include the granularity and inability to identify more specific mechanisms of injury such as motor vehicle crashes, gunshot wounds, or knife wounds. While most previous studies are focused in highly populated cities, this study involves multiple hospitals spread among multiple states therefore the distribution of the collected data may affect results. Additionally, hospitals may not have equal experiences as state and local responses to the COVID-19 pandemic have varied.

## Conclusion

Trauma volumes have dramatically changed during the COVID-19 pandemic with an initial decrease in March and a second decrease in November. Mechanism of injury has also seen dramatic changes with increases in penetrating trauma. Lastly, trauma mortality appears to have fluctuated with the COVID-19 pandemic. Further studies detailing these unique inflection points may help describe how human behavior affects trauma patients.
